# Combined Effects of *TGFB1* +869 T/C and +915 G/C Polymorphisms on Acute Rejection Risk in Solid Organ Transplant Recipients: A Systematic Review and Meta-Analysis

**DOI:** 10.1371/journal.pone.0093938

**Published:** 2014-04-04

**Authors:** Yu-Zheng Ge, Ran Wu, Tian-Ze Lu, Rui-Peng Jia, Ming-Hao Li, Xiao-Fei Gao, Xiao-Min Jiang, Xian-Bo Zhu, Liang-Peng Li, Si-Jia Tan, Qun Song, Wen-Cheng Li, Jia-Geng Zhu

**Affiliations:** 1 Center for Renal Transplantation, Nanjing First Hospital, Nanjing Medical University, Nanjing, China; 2 Department of Urology, Nanjing First Hospital, Nanjing Medical University, Nanjing, China; 3 Department of Cardiology, Nanjing First Hospital, Nanjing Medical University, Nanjing, China; 4 Department of General Surgery, Nanjing First Hospital, Nanjing Medical University, Nanjing, China; 5 Department of Cardiothoracic Surgery, Nanjing First Hospital, Nanjing Medical University, Nanjing, China; University of North Carolina School of Medicine, United States of America

## Abstract

**Background:**

Transforming growth factor-beta 1(TGF-β1) is involved in the development of acute rejection (AR) episodes in solid organ transplant recipients; and a number of studies have been conducted to investigate the combined effects of human TGF-β1 gene (*TGFB1*) +869 T/C and +915 G/C polymorphisms on AR risk. However, the results obtained are inconclusive.

**Methods:**

Eligible studies that investigated the haplotypic association between *TGFB1* +869 T/C and +915 G/C polymorphisms and AR risk were comprehensively searched in the PUBMED, EMBASE, China National Knowledge Infrastructure, and Wanfang Database. Statistical analyses were performed by using STATA 12.0 and Review Manager 5.0.

**Results:**

Fourteen eligible studies with 565 AR cases and 1219 non-AR cases were included. Overall, a significantly decreased risk was detected in patients carried with intermediate producer (IP) haplotypes (T/C G/C, T/T G/C, and C/C G/G) and/or low producer (LP) haplotypes (C/C G/C, C/C C/C, T/T C/C, and T/C C/C) compared with high producer (HP) haplotypes (T/T G/G and T/C G/G; IP vs. HP: OR = 0.75, 95% CI, 0.58–0.96, P _heterogeneity_  = 0.238; IP/LP vs. HP: OR  = 0.77, 95% CI, 0.61–0.98, P _heterogeneity_  = 0.144). In addition, subgroup analysis by transplant types demonstrated a similar association in patients receiving heart transplant (IP vs. HP: OR  = 0.32, 95% CI, 0.14–0.73, P _heterogeneity_  = 0.790; IP/LP vs. HP: OR  = 0.41, 95% CI, 0.20–0.85, P _heterogeneity_  = 0.320).

**Conclusions:**

The current meta-analysis and systematic review indicated that recipient *TGFB1* HP haplotypes were significantly associated with an increased risk for AR in solid organ transplant recipients, particularly patients receiving cardiac allograft.

## Introduction

Transforming growth factor-beta 1 (TGF-β1) is a multifunctional cytokine ubiquitously produced by a wide variety of cells, including T lymphocytes, monocytes, vascular endothelium, and renal tubular cells [1]. Functionally, TGF-β1 has been proven to be of fundamental importance in the development of various disorders [2], including coronary heart disease [3], human cancers [4], rheumatoid arthritis [5], asthma [6] and transplant rejection [7], [8]. In the setting of solid organ transplants, TGF-β1 has been conventionally recognized as a guardian against acute rejection (AR), as higher level of TGF-β1 in the graft tissue and serum was found in non-AR recipients than those suffering AR [9]–[11]. However, several novel lines of evidence have challenged the beneficial effects of TGF-β1 on transplant recipients [12], [13]. Although the functional role of TGF-β1 in AR initiation remains elusive, this cytokine is believed to exert pivotal and complicated functions in AR episodes.

The human TGF-β1 gene (*TGFB1*) is mapped on the chromosome 19q13.1–13.3 with seven exons and six introns, whose regulation and expression is influenced by the presence of single nucleotide polymorphisms (SNPs) [14]. Among these SNPs, +869 T/C (also known as rs1800470, T29C, or Leu10Pro) and +915 G/C (also termed as rs1800471, G74C, or Arg25Pro) polymorphisms in the first exon of *TGFB1* have been the focus of extensive researches and donor *TGFB1* +869 T/C polymorphism has been proven to be significantly associated with AR risk [15]. These two SNPs together result in nine potential inherited haplotypes, which could be categorized into three groups according to the *in vitro* production levels: high producer (HP) (T/T G/G and T/C G/G), intermediate producer (IP) (T/C G/C, T/T G/C, and C/C G/G) and low producer (LP) (C/C G/C, C/C C/C, T/T C/C and T/C C/C) [16], [17]. Since the first study conducted by Pelletier et al to evaluate the haplotypic association of *TGFB1* +869 T/C and +915 G/C polymorphisms with AR risk in kidney transplant recipients [18], numerous molecular epidemiological studies have been conducted in different solid organ transplants, including kidney transplants [19]–[26], liver transplants [9], [27], [28], and heart transplants [13], [29]. However, the results of these studies were inconclusive.

In this meta-analysis, we integrated the data from all eligible studies to explore 1) the combined effects of recipient *TGFB1* +869 T/C and +915 G/C polymorphisms on AR risk after solid organ transplantation and 2) the potential influence of covariants such as ethnicity, transplantation types, and immunosuppressive protocols.

## Materials and Methods

### Identification of eligible studies

This meta-analysis was conducted and reported in accordance with the PRISMA (Preferred Reporting Items for Systematic reviews and Meta-Analyses) guidelines (Checklist S1) [30]. To identify all eligible studies that investigated the haplotypic association of *TGFB1* +869 T/C and +915 G/C polymorphisms with AR risk in solid organ transplantation, a comprehensive electronic search of PUBMED, EMBASE, China National Knowledge Infrastructure (CNKI), and Wanfang databases was performed until November 29, 2013. To search and include as many related studies as possible, we applied various combinations of the following medical subject headings and key words: transforming growth factor beta-1, TGFβ-1, or *TGFB1*; acute rejection, early allograft outcome, or graft rejection; transplantation or organ transplantation; polymorphisms or variants. Furthermore, the reference lists of reviews and retrieved articles were manually screened for additional studies No restrictions were placed on language, and only published studies with full-text articles were included.

### Inclusion and exclusion criteria

The studies identified from the above mentioned databases were screened by two independent authors (Yu-Zheng Ge and Ran Wu) according to the following predesigned inclusion criteria: 1) case-control design; 2) evaluating the correlation of *TGFB1* haplotypes (at position +869 and +915) with AR risk; and 3) providing sufficient data to calculate the odds ratio (OR) and its corresponding 95% confidence interval (CI). When several studies with overlapping data were eligible, those with smaller sample size or less reliability were excluded. In addition, studies without detailed information were excluded, after the efforts to extract data from the original paper or contact the corresponding authors failed.

### Data extraction

Data of eligible studies were extracted by two reviewers (Yu-Zheng Ge and Tian-Ze Lu) independently and in duplicate according to the predesigned data-collection form. The following information was extracted: last name of first author, publication year, country of origin, ethnicity, transplantation type, immunosuppressive protocol, number of both ARs and non-ARs and phenotypic distribution in both groups. Different ethnic descents were categorized as Asian, Caucasian, African and Mixed (which included more than one ethnic descent). Transplantation types were characterized as renal, liver, and heart transplantation. Based on different calcineurin inhibitors (CNIs) within the immunosuppressive protocol, the studies were divided into two subgroups: Cyclosporine A (CsA) group and CsA/tacrolimus (FK506) group. Discrepancies occurring during the process of study inclusion and data extraction were resolved by discussion with a third reviewer (Wen-Cheng Li), and consensus on each item was achieved eventually.

### Statistical analysis

Crude OR and its corresponding 95% CI were used to assess the strength of association between the haplotypes of +869 T/C and +915 G/C polymorphisms in *TGFB1* gene and AR risk. The HP haplotypes (T/T G/G and T/C G/G) were used as the baseline for calculation of ORs in three different comparisons (LP vs. HP, IP vs. HP and LP/IP vs. HP). Stratified analyses were also conducted based on ethnicity, transplant types, and immunosuppressive protocols. The statistical significance of the pooled OR was assessed with the Z test, and P<0.05 was considered significant.

Chi-square based Q-test was performed to measure between-study heterogeneity, and the presence of heterogeneity was considered significant if P<0.10 [31]. When the heterogeneity was absent, the fixed-effect model (Mantel-Haenszel method) was used to pool the data from different studies [32]; otherwise, the random-effects model (DerSimonian and Laird method) was applied [33]. Sensitivity analyses were conducted to identify the effect of individual study on pooled results and to test the reliability of results by deleting a single study each time [34]. To determine the presence of publication bias, both Begg's funnel plot and Egger's linear regression test were conducted, and P<0.05 was considered significant [35], [36].

All statistical analyses were performed with STATA software (version 12.0; Stata Corporation, College Station, Texas, USA) and Review Manager (version 5.0; Cochrane Collaboration, Oxford, UK).

## Results

### Characteristics of eligible studies

**Figure 1:**
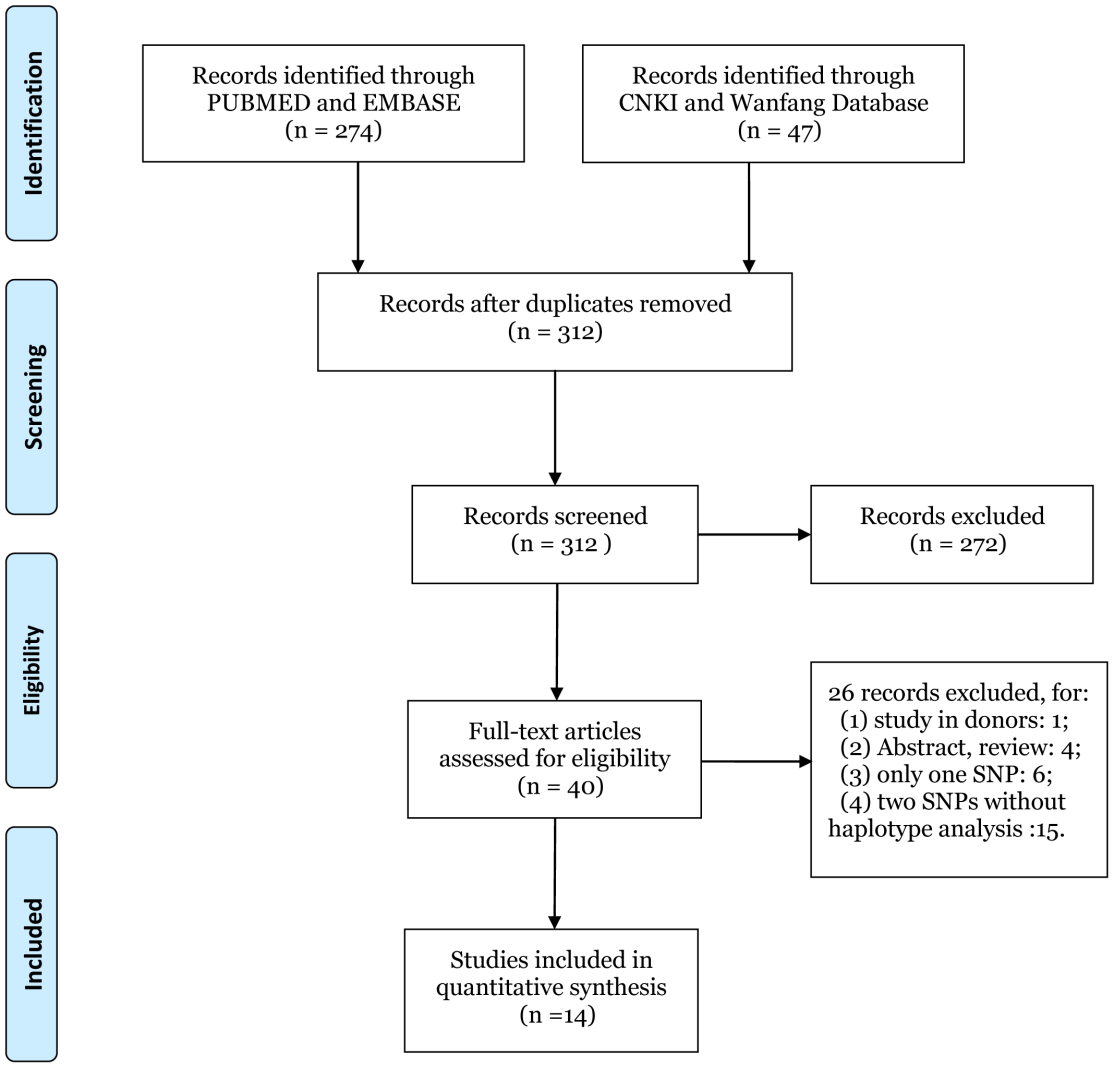
Flow diagram for study selection. Description: A total of 14 studies were included in this meta-analysis and systematic review after a comprehensive study selection.

Fourteen eligible articles were identified according to the predesigned selection criteria, and the detailed screening process was shown in Figure 1. Among the 14 eligible studies, 9 were renal transplantation[18]–[26], 3 were liver transplantation [9], [27], [28], and 2 were heart transplantation[13], [29]. As for ethnicity, 7 were studies of Caucasians [9], [22], [23], [27], [28], [37], 3 studies were of Asians [19]–[21], and 4 studies of mixed ethnicity [13], [18], [26], [29]. Regarding the immunosuppressive protocols, patients were uniformly prescribed with CsA in 5 studies[18]–[20], [23], [29] while in the remaining 9 studies were applied with either CsA or FK506[9], [13], [21], [22], [24]–[28] (Table 1).

**Table 1:** Characteristics and haplotypes distribution of eligible studies included in the meta-analysis.

Author	Year	Country	Ethnicity	Transplant types	CNIs	AR/non-AR	AR cases			Non-AR		
							HP	IP	LP	HP	IP	LP
Pelletier	2000	USA	Mixed	RTx	CsA	25/46	7	17	1	15	28	3
Tambur	2001	USA	Caucasian	LTx	CsA/FK506	33/30	24	8	1	16	12	2
Tian	2001	China	Asian	RTx	CsA	19/72	16	3	0	56	16	0
Tian	2002	China	Asian	RTx	CsA	26/89	23	3	0	68	21	0
Gu	2003	China	Asian	RTx	CsA/FK506	23/74	19	4	0	66	7	1
Karasu	2004	Turkey	Caucasian	LTx	CsA/FK506	26/17	19	7	0	11	6	0
Gourley	2004	USA	Mixed	HTx	CsA	42/50	32	6	4	32	17	1
Lacha	2005	Czech	Caucasian	RTx	CsA/FK506	117/228	33	65	19	82	121	25
Di Filippo	2006	USA	Mixed	HTx	CsA/FK506	39/72	36	3	0	54	16	2
Gómez-Mateo	2006	Spain	Caucasian	LTx	CsA/FK506	42/108	32	9	1	74	27	7
Canossi	2007	Italy	Caucasian	RTx	CsA	25/61	19	5	1	37	21	3
Kocierz	2011	Poland	Caucasian	RTx	CsA/FK506	49/150	43	5	1	113	32	5
Seyhun	2012	Turkey	Caucasian	RTx	CsA/FK506	19/71	14	4	1	39	17	15
Dhaouadi	2013	Tunisia	Mixed	RTx	CsA/FK506	80/151	55	17	8	105	38	8

Abbreviations: RTx, renal transplantation; LTx, liver transplantation; HTx, heart transplantation; CNIs, calcineurin inhibitors; CsA, cyclosporine A; FK506, tacrolimus; AR, acute rejection; LP, low producer; IP, intermediate producer; HP, high producer.

### Quantitative data synthesis

**Figure 2:**
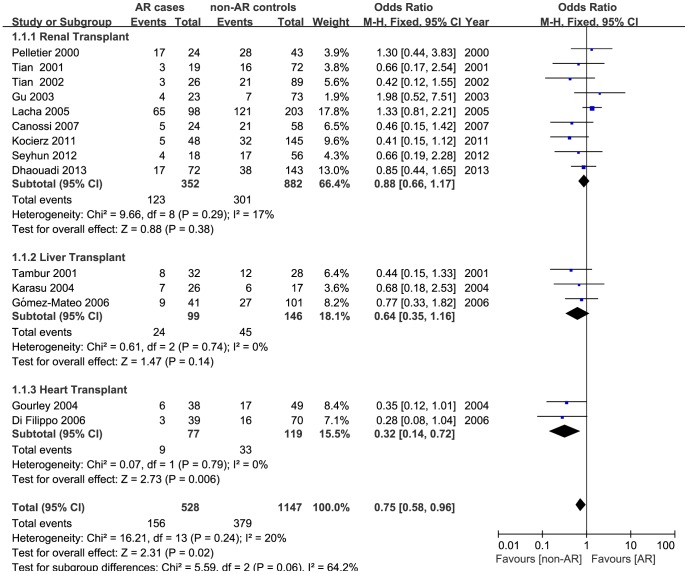
Forest plot for risk of acute rejection associated with *TGFB1* haplotypes (IP vs. HP) stratified by transplant types. For each study, the estimate of OR and its 95% CI is plotted with a *box* and a *horizontal line*. *Filled diamond* pooled OR and its 95% CI.

**Figure 3:**
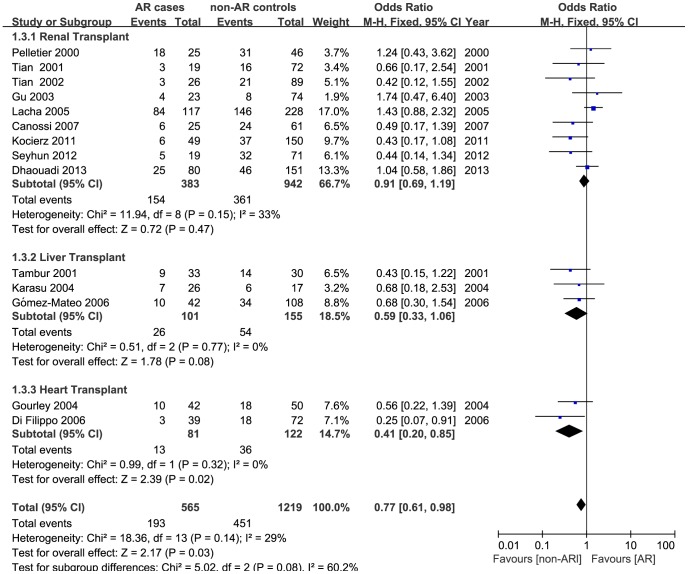
Forest plot for risk of acute rejection associated with *TGFB1* haplotypes (LP/IP vs. HP) stratified by transplant types. For each study, the estimate of OR and its 95% CI is plotted with a *box* and a *horizontal line*. *Filled diamond* pooled OR and its 95% CI.

A total of 14 studies including 565 AR and 1219 non-AR cases were identified to assess the haplotypic association of *TGFB1* +869 T/C and +915 G/C polymorphisms with AR risk of solid organ transplantation. Overall, a significantly decreased risk was detected in two comparisons (IP vs. HP: OR  = 0.75, 95% CI, 0.58–0.96, P _heterogeneity_  = 0.238, Figure 2; IP/LP vs. HP: OR  = 0.77, 95% CI, 0.61–0.98, P _heterogeneity_  = 0.144, Figure 3). Subgroup analyses based on ethnicity, transplant types, and immunosuppressive protocols were subsequently conducted, and the results demonstrated a remarkably decreased risk in heart transplant recipients (IP vs. HP: OR  = 0.32, 95% CI, 0.14–0.73, P _heterogeneity_  = 0.790, Figure 2; IP/LP vs. HP: OR  = 0.41, 95% CI, 0.20–0.85, P _heterogeneity_  = 0.320, Figure 3) and in patients uniformly administrated with CsA (IP vs. HP: OR = 0.57, 95% CI, 0.34–0.94, P _heterogeneity_ = 0.491). Table 2 represents the strength of association between *TGFB1* haplotypes (at position +869 and +915) and AR risk in transplant recipients.

**Table 2:** Stratification analyses of combined effects of *TGFB1* +869 T/C and +915 G/C polymorphisms on acute rejection risk.

Category	LP vs. HP				IP vs. HP				LP/IP vs. HP			
	N^a^	OR(95% CI)	P^b^	P^c^	N^a^	OR(95% CI)	P^b^	P^c^	N^a^	OR(95% CI)	P^b^	P^c^
Total	11	1.10(0.71,1.71)	0.665	0.373	14	**0.75(0.58,0.96)** d	**0.021** d	0.238	14	**0.77(0.61,0.98)** d	**0.030** d	0.144
**Ethnicity**												
Asian	1	1.14(0.05,29.0)	0.938	—	3	0.76(0.36,1.60)	0.466	0.242	3	0.74(0.35,1.55)	0.425	0.303
Caucasian	6	0.93(0.54,1.61)	0.807	0.165	7	0.80(0.58,1.10)	0.174	0.251	7	0.78(0.58,1.06)	0.112	0.094
Mixed	4	1.58(0.72,3.49)	0.258	0.507	4	0.65(0.42,1.03)	0.064	0.163	4	0.77(0.51,1.16)	0.214	0.160
**Transplantation Types**												
RTx	7	1.23(0.75,2.01)	0.406	0.401	9	0.88(0.66,1.17)	0.377	0.290	9	0.91(0.69,1.19)	0.473	0.154
LTx	2	0.33(0.07,1.69)	0.185	0.996	3	0.64(0.35,1.16)	0.142	0.737	3	0.59(0.33,1.06)	0.076	0.774
HTx	2	1.50(0.34,6.57)	0.594	0.180	2	**0.32(0.14,0.73)** d	**0.006** d	0.790	2	**0.41(0.20,0.85)** d	**0.017** d	0.320
**Immunosuppressive protocol**												
CsA	3	1.37(0.41,4.59)	0.607	0.462	5	**0.57(0.34,0.94)** d	**0.027** d	0.491	5	0.62(0.38,1.01)	0.055	0.694
CsA/FK506	8	1.07(0.66,1.71)	0.793	0.229	9	0.82(0.62,1.09)	0.169	0.194	9	0.83(0.64,1.08)	0.162	0.059

Abbreviations: RTx, renal transplantation; LTx, liver transplantation; HTx, heart transplantation; CsA, cyclosporine A; FK506, tacrolimus; LP, low producer; IP, intermediate producer; HP, high producer.

—, cannot be calculated;

a Number of eligible studies;

b P value of Z-test for significance of OR;

c P value of Q-test for heterogeneity test;

d **Statistically significant results (in bold).**

### Heterogeneity test and sensitivity analysis

No significant between-study heterogeneity was observed in this meta-analysis (Table 2). Sensitivity analysis was also performed to explore the potential influence of each individual study on the overall results by deleting one single study each time from the pooled analysis. NO substantial change was demonstrated in the overall studies, indicating that no individual study could affect the pooled OR significantly (data not shown).

### Publication bias

To examine the publication bias of the currently available literature, both Begg's funnel plot and Egger's test were conducted. The shape of the funnel plots did not reveal any evidence of obvious asymmetry in all comparison models (Figures S1, S2, S3). Then, the Egger's test was used to provide statistical evidence for funnel plot symmetry. The results also did not show any evidence of publication bias (Table S1).

## Discussion

Organ transplantation has been recommended as the most optimal treatment choice for patients suffering end-stage disease. However, AR still remains a crucial determining factor which influences the short-term function and long-term outcome of both recipients and allografts [38]–[40]. To the best of our knowledge, this is the first meta-analysis focusing on the combined effects of *TGFB1* +869 T/C and +915 G/C polymorphisms on AR risk. In the current study, we provide evidence that patients carried with *TGFB1* HP haplotypes (T/T G/G and T/C G/G) are more likely to suffer from AR after solid organ transplantation (specifically heart transplantation), which could be utilized to identify patients predisposed to AR and potentially benefiting from tailored immunosuppressive protocol.

After pooling the data from 14 eligible studies, we shown that patients with *TGFB1* IP and/or LP haplotypes were less likely to suffer from AR than those with HP haplotypes (IP vs. HP: OR  = 0.75, 95% CI, 0.58–0.96; IP/LP vs. HP: OR  = 0.77, 95% CI, 0.61–0.98). Considering that ethnic background could influence the frequency of genotypes and haplotypes in some context, we conducted subgroup analysis based on ethnicities but failed to detect any significant association. Further stratified analysis by transplant types demonstrated a remarkably decreased AR risk for heart transplant recipients carried with *TGFB1* IP and/or LP haplotypes, which was in agreement with the study conducted in a cohort of 111 pediatric cardiac transplant recipients [13]. With respect to various CNIs applied in suppressive protocols, CsA and FK506 have been proven to influence the serum level of TGF-β1 differentially [41], [42]; thus, we intended to investigate the various impacts of different CNIs on the relationship between the *TGFB1* haplotypes and AR risk. However, FK506 has never been administrated as a unique CNI in any included studies. Therefore, we divided the studies into two subgroups: CsA arm and CsA/FK506 arm, and then conducted a subgroup analysis. As a result, patients with *TGFB1* IP haplotypes were less likely to be affected by AR in CsA arm (IP vs. HP: OR  = 0.57, 95% CI, 0.34–0.94), which could be interpreted as a clue that the recipients with *TGFB1* IP haplotypes can benefit from CsA after organ transplantation.

TGF-β1 is a pleiotropic and multifunctional cytokine with immunosuppressive and fibrogenic properties, which may play a central role in both the initiation and propagation of AR and chronic rejection (CR) [7], [8]. The expression of TGF-β1 has been proven to be regulated by the two SNPs (+869 T/C and +915 G/C polymorphisms), whose combination could be divided into three groups (LP, IP, and HP). However, the results obtained have challenged the conventional concept that TGF-β1 may inhibit the initiation of AR episodes. Our team previously demonstrated that donor *TGFB1* +869 T/C HP genotype (TT) was significantly associated with decreased AR risk [15]. Considering the obvious discrepancies, several questions have been raised: 1) The impact of various haplotypes of *TGFB1* +869 T/C and +915 G/C polymorphisms on actual production levels of TGF-β1. The effects of the nine potential haplotypes on TGF-β1 expression levels have been previously studied *in vitro* and among patients without organ transplant [16], [43], [44]. However, no significant association between *TGFB1* SNPs and TGF-β1 plasma levels or intragraft mRNA levels was detected in a cohort of renal transplant recipients [45]. 2) The influence of various immunosuppressive protocols on the expression level of TGF-β1. CsA and FK506 were found to influence the serum level of TGF-β1 differentially [41], [42], [45], while the impact of other immunosuppressive regimens still remains elusive. 3) The potential effects of donor and/or donor-recipient pair *TGFB1* haplotypes on AR risk. The current study is confined to recipient SNPs; however, in some context, donor polymorphisms may contribute much more to AR risk than recipient SNPs [15], [46]. 4) The exact etiology of AR episodes and the biological functions of TGF-β1 in the development of AR still remain unclear.

The current meta-analysis focused on the combined effects of *TGFB1* +869 T/C and +915 G/C polymorphisms rather than one single SNP on AR risk [15], which could help to derive a precise estimation of the roles of *TGFB1* SNPs in the development of AR episodes. However, several limitations should be considered when interpreting the results. First, considering the unavailability of other detailed information, we did not conduct stratified analysis based on some cofactors such as follow-up time, gender, age, panel reactive antibodies level, human leukocyte antigens mismatch and donor source, which may influence the results. Second, the limited number of AR cases and non-AR cases may lead to a relatively small power. Third, only published studies with sufficient data were included, thus, publication bias may have occurred even though results of both Begg's test and Egger's test did not detect it. Last but not least, the meta-analysis is retrospective due to the methodological limitations.

In summary, this meta-analysis suggested that recipient *TGFB1* HP haplotypes of +869 T/C and +915 G/C polymorphisms (T/T G/G and T/C G/G) might be a possible genetic susceptibility locus for AR after solid organ transplantation, which could be utilized to identify patients predisposed to AR and potentially benefiting from personalized immunosuppressive protocol. In addition, monitoring TGFB1 could help manage CR to some extent, as TGFB1 triggers fibrogenesis linked to chronic rejection (CR). Further well-designed and unbiased studies with larger sample size, diverse ethnicities, donor-recipient pairing and various applications of CNIs should be conducted to verify our findings. Furthermore, functional studies of *TGFB1* gene polymorphism are warranted to understand the underlying mechanisms.

## Supporting Information

Figure S1 **Begg's funnel plot for publication bias test (LP vs. HP for** ***TGFB1*** **haplotypes).**
(TIF)

Figure S2 **Begg's funnel plot for publication bias test (IP vs. HP for** ***TGFB1*** **haplotypes).**
(TIF)

Figure S3 **Begg's funnel plot for publication bias test (LP/IP vs. HP for** ***TGFB1*** **haplotypes).**
(TIF)

Checklist S1 **PRISMA checklist.**
(DOC)

Table S1 **Statistical analyses of publication bias for** ***TGFB1*** **haplotypes at +869 T/C and +915 G/C polymorphisms.**
(DOC)
