# Supplementary figures and images for: Combined Effects of TGFB1 +869 T/C and +915 G/C Polymorphisms on Acute Rejection Risk in Solid Organ Transplant Recipients: A Systematic Review and Meta-Analysis
Source: PLoS One. 2014 Apr 4;9(4):e93938. doi: 10.1371/journal.pone.0093938 (PMC3976347; doi:10.1371/journal.pone.0093938)

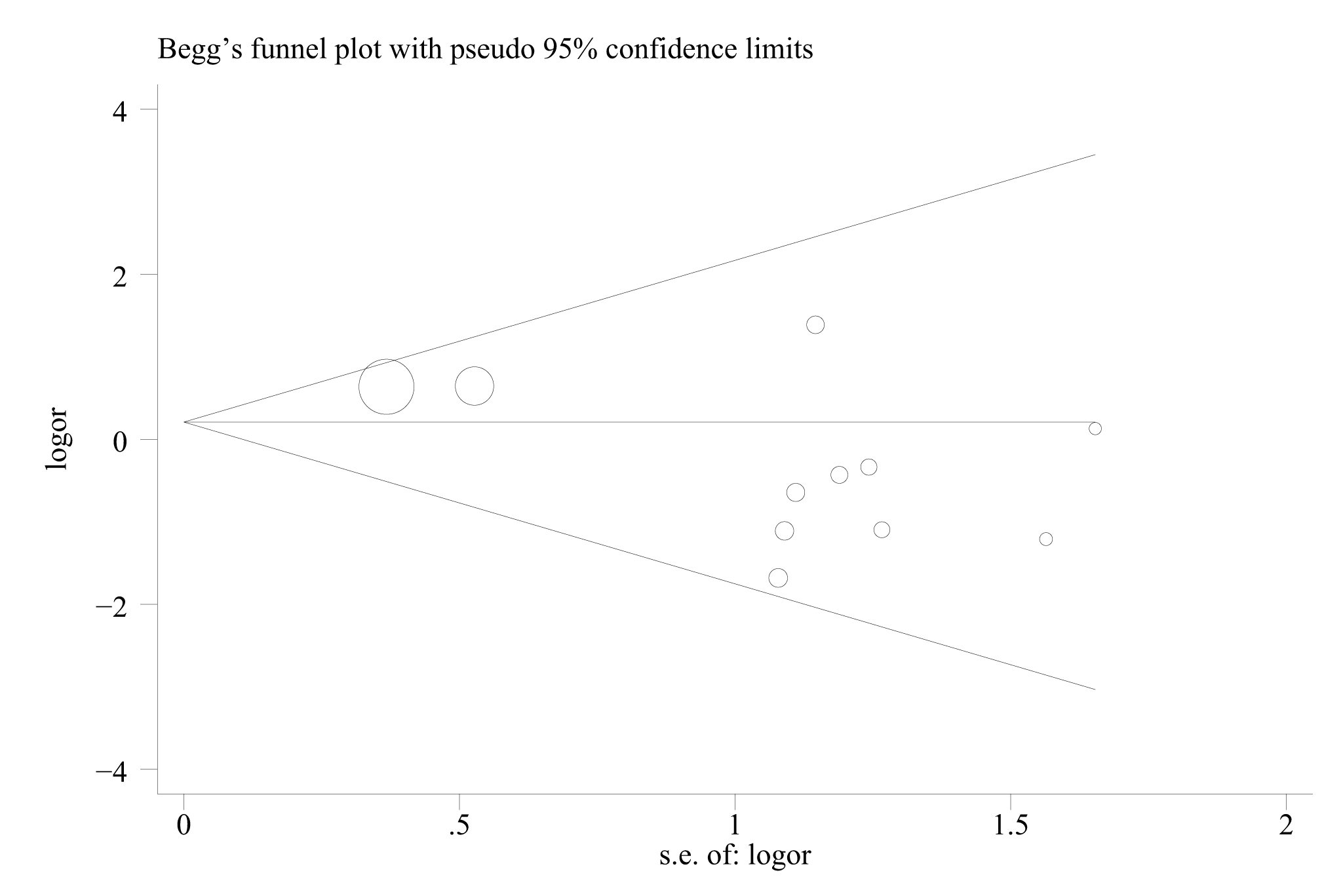

Supplement: Figure S1 — Begg's funnel plot for publication bias test (LP vs. HP for TGFB1 haplotypes). (TIF) [file pone.0093938.s001.tif]

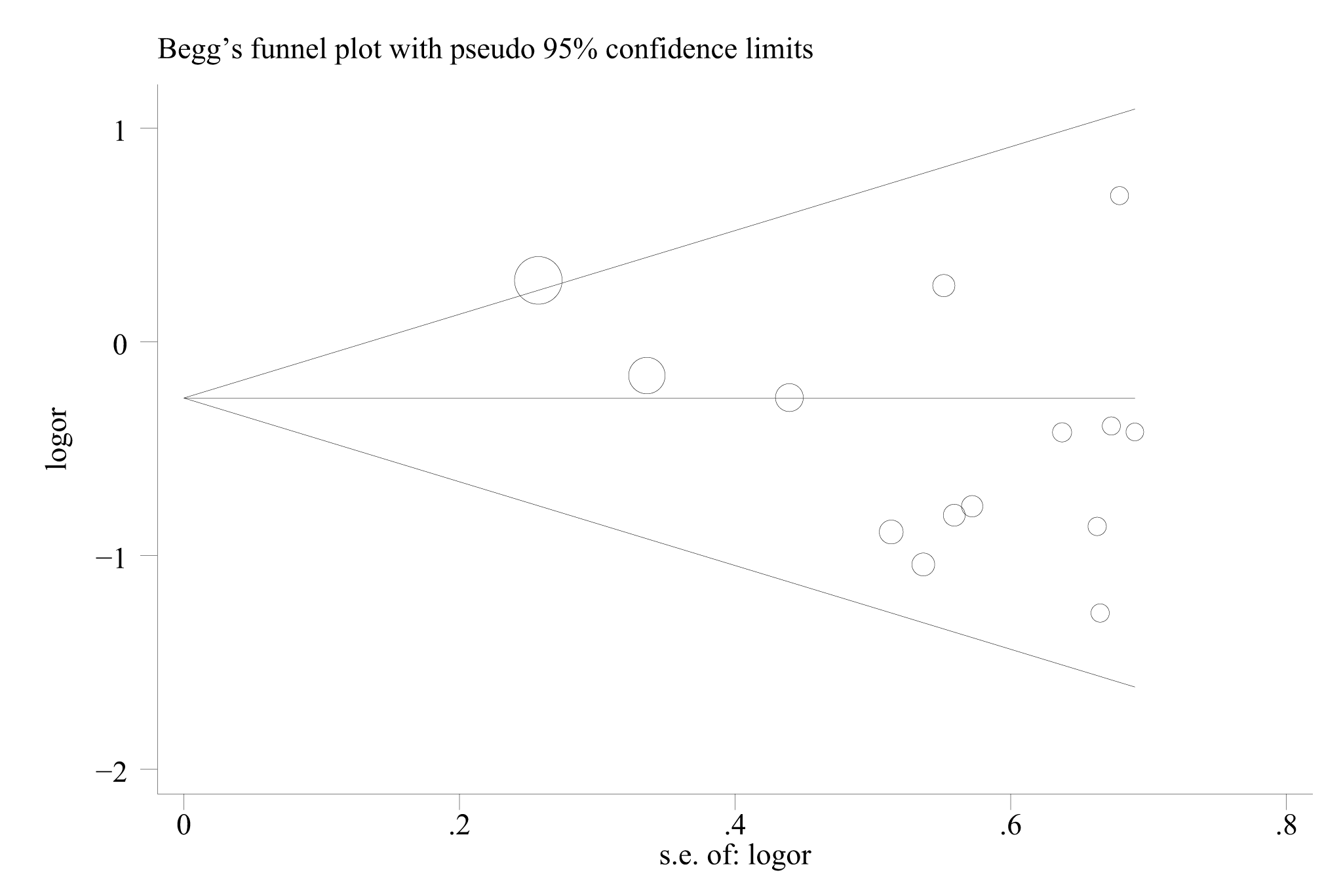

Supplement: Figure S2 — Begg's funnel plot for publication bias test (IP vs. HP for TGFB1 haplotypes). (TIF) [file pone.0093938.s002.tif]

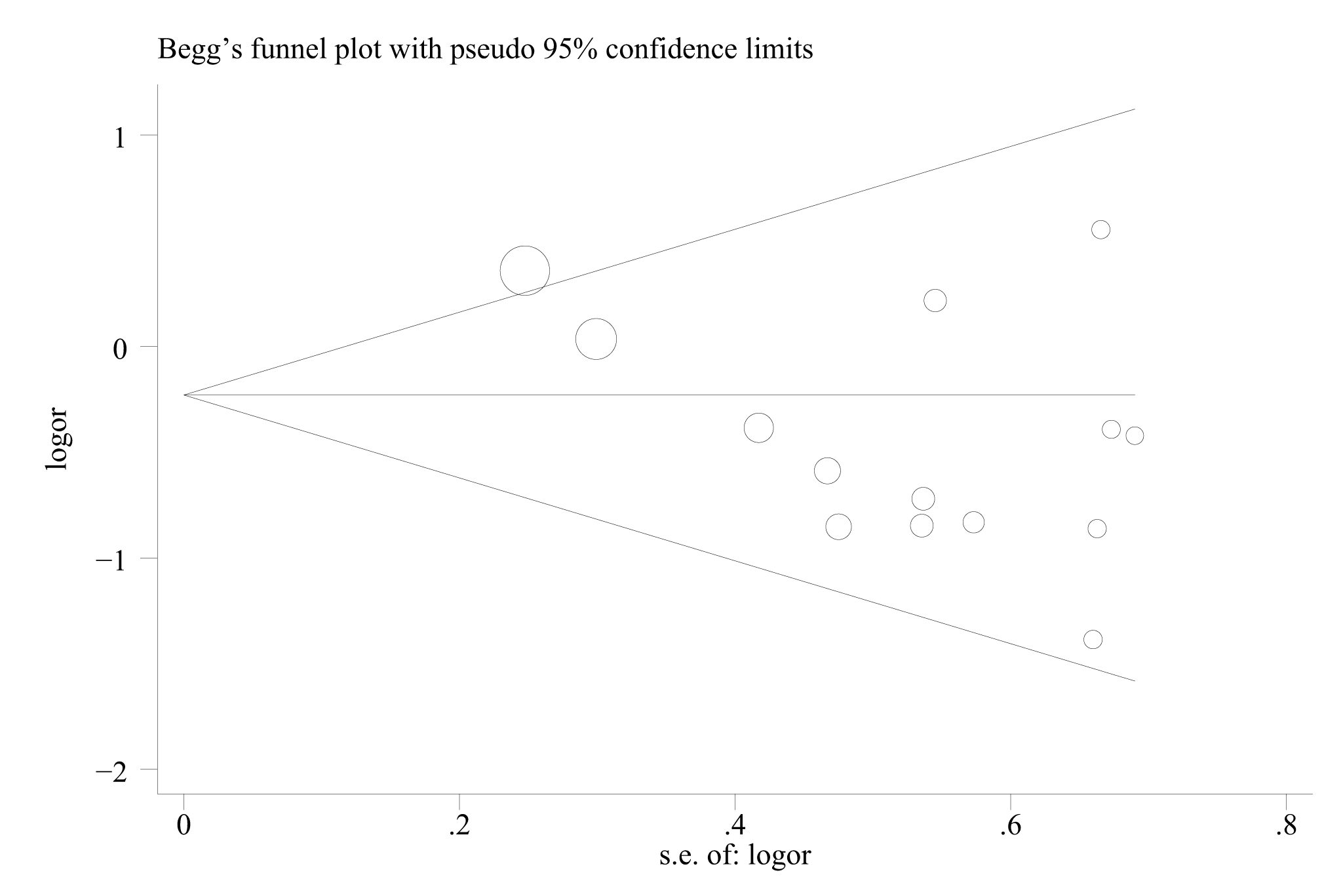

Supplement: Figure S3 — Begg's funnel plot for publication bias test (LP/IP vs. HP for TGFB1 haplotypes). (TIF) [file pone.0093938.s003.tif]
