# Supplementary material for: Combined Effects of TGFB1 +869 T/C and +915 G/C Polymorphisms on Acute Rejection Risk in Solid Organ Transplant Recipients: A Systematic Review and Meta-Analysis
Source: PLoS One. 2014 Apr 4;9(4):e93938. doi: 10.1371/journal.pone.0093938 (PMC3976347; doi:10.1371/journal.pone.0093938)
Supplement: Table S1 — Statistical analyses of publication bias for TGFB1 haplotypes at +869 T/C and +915 G/C polymorphisms. (DOC) [file pone.0093938.s005.doc]

Table S1. Statistical analyses of publication bias for *TGFB1* haplotypes at +869 T/C and +915 G/C polymorphisms

| Category | LP vs. HP | IP vs. HP | LP/IP vs. HP |
| --- | --- | --- | --- |
| Begg’s test (P) | 0.876 | 0.661 | 0.584 |
| Egger’s test (P) | 0.119 | 0.130 | 0.111 |

LP, low producer; IP, intermediate producer; HP, high producer.
